# Supplementary material for: Feasibility of a social protection linkage program for individuals at-risk for tuberculosis in Uganda
Source: PLOS Glob Public Health. 2023 Dec 8;3(12):e0002122. doi: 10.1371/journal.pgph.0002122 (PMC10707648; doi:10.1371/journal.pgph.0002122)
Supplement: S1 Table — Characteristics reflect data collected in May-December 2021. (DOCX) [file pgph.0002122.s001.docx]

**S1 Table.** Characteristics and dates of implementation of government-supported social protection programs included in the linkage program.

| **Scheme** | **Program characteristics and eligibility** | **Benefits offered** | **Dates of implementation** |
| --- | --- | --- | --- |
| **Youth Livelihood Program (YLP)(1)** | - **Intent:** To harness social-economic potential and increase self-employment opportunities and income level among Ugandan youth - **Population:** Men/Women, 18-30 years - **Special groups/requirements**: Unemployed/underemployed, poor youth - **Frequency of distribution:** Quarterly - **Distribution level:** Group - **Typical loan amount:** up to 12,500,000 Ugandan Shillings - **Group size:** 10-15 | Loan | 2014-present |
| **Uganda Women Entrepreneurship Program (UWEP)(2,3)** | - **Intent:** To improve access to financial services for women, to equip them with skills for enterprise growth, value addition, and marketing of their products and services, and to increase participation of women in business development, increase their incomes, livelihood security, and quality of life - **Population:** Women, 18-79 years - **Special groups/requirements:** Vulnerable populations including unemployed, women heading households, single young mothers, widows and gender-based violence (GBV) survivors - **Frequency of distribution:** Quarterly - **Distribution level:** Group - **Typical loan amount:** 5,000,000 – 12,000,000 Ugandan Shillings - **Group size:** 5-10 | Loan | 2015-present |
| **Emyooga(4)** | - **Intent:** To transform 68% of homesteads from subsistence to market-oriented production with the overall objective of promoting job creation and improving household incomes - **Population:** Men/Women, ≥18 years - **Special groups/requirements**: Beneficiaries must save within their group prior to accessing benefits - **Frequency of distribution:** One-time - **Distribution level:** Group - **Distribution amount:** 30,000,000 Ugandan Shillings - **Group size:** 7-30 | Loan | 2019-present |
| **Parish Development Model (PDM)(5)** | - **Intent:** To organize and deliver public and private sector interventions for wealth creation and employment generation at the parish level (lowest economic planning unit) to ensure support for more Ugandans in order to increase their demand for goods and services - **Population:** Men/Women, ≥18 years - **Special groups/requirements**: None - **Frequency of distribution:** One-time distribution - **Distribution level:** Group - **Distribution amount:** 100,000,000 Ugandan Shillings/parish - **Group size:** 10-30 | Loan | 2022-present |
| **Social Assistance Grant for Empowerment (SAGE)(6)** | - **Intent:** To enable senior citizens to access basic services, and to start income generating activities - **Population:** Men/Women, ≥80 years - **Special groups/requirements**: None - **Frequency of distribution:** Monthly - **Distribution level:** Individual - **Distribution amount**: 25,000 Ugandan Shillings | Grant | 2015-present |
| **People with Disabilities (PWD)(7)** | - **Intent:** To promote equal opportunities for enhanced empowerment, participation and protection of rights of PWDs irrespective of gender, age and type of disability. This is in recognition that PWDs can perform to their full potential given the same conditions and opportunities irrespective of their social, economic and cultural backgrounds - **Population:** Men/Women, ≥18 years - **Special groups/requirements:** Adults living with disabilities - **Frequency of distribution:** Quarterly - **Distribution level:** Group - **Distribution amount:**1,000,000-5,000,000 Ugandan Shillings/group - **Group size:** 5-15 | Grant | 2019-present |
| **Operation Wealth Creation (OWC)(8)** | - **Intent:** To distribute farm inputs to farmers and to coordinate government Ministries, Departments, and Agencies for improved service delivery and to efficiently facilitate national socio-economic transformation, with a focus on raising household incomes and wealth creation by transforming subsistence farmers into commercial farmers. - **Population:** Men/Women, ≥18 years - **Special groups/requirements:** Land ownership - **Frequency of distribution:** Quarterly - **Distribution level:** Individual | Agricultural inputs | 2013-present |

Characteristics reflect data collected in May-December 2021.

**REFERENCES**

1. Youth Livelihood Programme (YLP) Programme Document. Kampala, Uganda: Ministry of Gender, Labour and Social Development; 2013 Dec.

2. Uganda Women Entrepreneurship Programme (UWEP) Programme Document. Kampala, Uganda: Ministry of Gender, Labour and Social Development; 2018 May.

3. Revised Guidelines for the Implementation of the Uganda Women Entrepreneurship Programme (UWEP) Programme. Kampala, Uganda: Ministry of Gender, Labour and Social Development; 2019 May.

4. Presidential Initiative on Wealth and Job Creation (Emyooga) Draft Operational Guidelines. Kampala, Uganda: Ministry of Finance, Planning and Economic Development;

5. Implementation Guidelines for the Parish Development Model. Kampala, Uganda: Ministry of Local Government; 2022 Feb.

6. The Senior Citizens Grant (SCG) Stakeholders’ Handbook: How SAGE Works in the Community. Kampala, Uganda: Expanding Social Protection Programme, Ministry of Gender, Labour and Social Development; 2022 May.

7. Guidelines on the Special Grant for Persons with Disabilities. Kampala, Uganda: Ministry of Gender, Labour and Social Development; 2020 Jan.

8. Operation Wealth Creation [Internet]. [cited 2023 Apr 19]. Available from: https://owc.go.ug/index.html
