# Supplementary material for: Feasibility of a social protection linkage program for individuals at-risk for tuberculosis in Uganda
Source: PLOS Glob Public Health. 2023 Dec 8;3(12):e0002122. doi: 10.1371/journal.pgph.0002122 (PMC10707648; doi:10.1371/journal.pgph.0002122)
Supplement: S2 Table — CDO = community development office; HC = health center; IQR = interquartile range. (DOCX) [file pgph.0002122.s002.docx]

**S2 Table. Screening and referral metrics among linked participants by health center.**

| **Characteristic** | **Health Center (HC) 1** | **HC 2** | **HC 3** | **HC 4** |
| --- | --- | --- | --- | --- |
| Screened | 332 | 128 | 138 | 257 |
| Eligible and referred, n (%) | 281 (84.6) | 121 (94.5) | 44 (31.9) | 184 (71.6) |
| Linked to subcounty CDO, n (%) | 225 (80.1) | 93 (76.9) | 23 (52.3) | 45 (24.5) |
| Enrolled into social protection scheme, n (%) | 109 (48.4) | 8 (8.6) | 3 (13.0) | 2 (4.4) |
| Days to present to subcounty CDO, Median (IQR) | 2 (0-6) | 0 (0-0) | 3 (0-15) | 0 (0-1) |
| Transportation reimbursement among linked, n (%) | 212 (94.2) | 85 (91.4) | 22 (95.7) | 43 (95.6) |

CDO=community development office; HC=health center; IQR=interquartile range
