# Supplementary material for: Feasibility of a social protection linkage program for individuals at-risk for tuberculosis in Uganda
Source: PLOS Glob Public Health. 2023 Dec 8;3(12):e0002122. doi: 10.1371/journal.pgph.0002122 (PMC10707648; doi:10.1371/journal.pgph.0002122)
Supplement: Attachment — Submitted filename: PLoS GH Response to Reviewers.docx [file pgph.0002122.s005.docx]

| UNIVERSITY OF CALIFORNIA, SAN FRANCISCO  berkeley • DAVIS • IRVINE • LOS ANGELES • RIVERSIDE • SAN DIEGO • SAn francisco |  | SANTA BARBARA • SANTA CRUZ |
| --- | --- | --- |

Priya Shete, M.D., M.P.H	Division of Pulmonary and Critical Care Medicine

Associate Professor of Medicine & Epidemiology	San Francisco General Hospital, Room 5K1

Co-Director, UCSF Implementation Science Training Program	1001 Potrero Avenue

Director, Collaborative on Pandemic Recovery and Readiness Research	San Francisco, CA 94110

Clinical Core Director, UCSF Center for Tuberculosis	Phone: 628-206-4694 | Fax: 628-206-8367

Email: [priya.shete@ucsf.edu](mailto:priya.shete@ucsf.edu)

August 26, 2023

Dear Dr. Gao and Editorial Colleagues,

Thank you very much for the opportunity to revise and resubmit our manuscript entitled “Feasibility of a social protection linkage program for individuals at-risk for tuberculosis in Uganda” (PGPH-D-23-01088) for consideration in PLOS Global Public Health. We are grateful for the reviews received and present below our responses and a summary of changes in response to Reviewer feedback.

We look forward to your response.

Sincerely,

Priya B. Shete, MD, MPH

**Summary of Review**

1. **In the ethics statement in the Methods, you have specified that verbal consent was obtained. Please provide additional details regarding how this consent was documented and witnessed, and state whether this was approved by the IRB.**

**Response:** We have provided additional information about the verbal consent process on page 9 of the manuscript; edits are also included below:

“*Verbal consent was obtained for participation in surveys by an experienced study staff member trained in human subjects research. Study staff members called participants and used an IRB-approved verbal consent form script to administer the consent form. Participants’ responses were audio recorded using REDCap.”*

2. **Please amend your detailed Financial Disclosure statement. This is published with the article. It must therefore be completed in full sentences and contain the exact wording you wish to be published.**
   - a. State the initials, alongside each funding source, of each author to receive each grant.
   - b. State what role the funders took in the study. If the funders had no role in your study, please state: “The funders had no role in study design, data collection and analysis, decision to publish, or preparation of the manuscript.”
   - c. If any authors received a salary from any of your funders, please state which authors and which funders.

**Response:** Thank you for this comment. We have revised the Financial Disclosure statement in the submission system. It now reads:

This work was supported by the UCSF-Gladstone Center for AIDS Research Mentored Science Award in HIV/AIDS Research (grant NIH P30 AI027763 Award A120163): PS; the Parker B. Francis Fellowship (grant P0523744): PS; and the US National Heart, Lung, and Blood Institute (grant NIH/NHLBI R01AI121144): AC. The funders had no role in study design, data collection and analysis, decision to publish, or preparation of the manuscript.

**Review Comments to the Author**

**Reviewer #1**

1. **Lines 137-139: Were people in other formal employment eligible for a social protection program?**

**Response:** Being a government worker in a salaried position was a main exclusion criterion of government-sponsored social protection programs included in this pilot study – for simplicity for the health workers implementing these eligibility criteria, we included this as criteria for inclusion in the study for all clients evaluated. All other people in formal employment were eligible for social protection programs as it was not an eligibility requirement for any other social protection program included in the evaluation.

2. **Lines 155-157: Was screening conducted by VHT based on the inclusion criteria described on line 137-139?**

**Response:** Yes, screening for eligibility criteria mentioned in the section “Eligibility criteria and study population” was conducted by routine VHTs at the health facility; we have edited the lines to indicate “The VHT assessed all persons under evaluation for TB for eligibility criteria described above and informed each person whether they were likely eligible for a social protection program based on results of the screening.”

3. **Table 1: Please include the denominator for results shown.**

**Response:** Thank you for this comment – we have added the denominator of N=630 at the top of the table.

4. **Lines 255-259: This sentence should appear earlier in the results, and in this section before presenting the survey results, the authors should include data and reasons for non-enrolment for the 264 who were not enrolled.**

**Response:** We agree with this suggestion and have moved this sentence, which was previously included as part of the caption for figure 1, to earlier in the body of the manuscript on lines 237-240.

5. **Lines 278 - 280: Consider rephrasing this sentence for clarity. It's unclear where the denominator 14 arises from, is this from the 97 participants who were surveyed? Also unclear what the total number out of 97 were actually enrolled in the program.**

**Response:** Thank you for the opportunity to clarify. We have edited the paragraph on page 13 in several places to address the comments above – please see below:

*“We compared self-reported data regarding social protection program referral, linkage and enrollment between HC and CDO logbooks. All survey participants (n=97) were found eligible for CDO referral, 60 (65%) presented to the CDO to register for a program, but ultimately only 14 (14%) were enrolled into a scheme. Four survey participants did not realize they had been found eligible and were referred by health center staff to the CDO for social protection enrollment. Eight survey participants whose logbook data reflected social protection program enrollment self-reported that they were not enrolled, while 9 survey participants self-reported that they were enrolled into a program when logbook data indicated that they were not. Among the 60 survey participants who presented to the CDO to register, 46 were not enrolled into a program (77%), Most of these participants (n=25; 54%) reported that they did not know the reason for why they were not enrolled (****Fig 1****). 63% (n=61) of all survey participants indicated that they would have not sought out a social protection program without this linkage program.”*

6. **Lines 281: What is the denominator for 54% based on?**

**Response:** Thank you for this comment – we have clarified in the text (please see response to comment directly above) to indicate that the 54% is among survey participants who successfully presented to the CDO but were ultimately not enrolled into a program (n=46). Please see lines 290-293 for updated text addressing this comment.

7. **Lines 311 - 316: Appears to be a repetition of results and should be in the results section.**

**Response:** Thank you for this comment. Indeed, these data are already reported in the results section, including in the text and in Table 3. We have edited this section to reduce the repetition of results and instead summarize what was found without reference to specific results. Please see edited text on lines 323-327, pasted below:

*“Surveys revealed many participants engaged in negative financial coping strategies, called dissaving, in order manage the costs of TB associated care seeking and symptoms, and that a vast majority faced unmet social needs including access to basic needs and services including food, housing, and medical care (****Table 3****).”*

8. **Lines 328-330: It's unclear what the 20% is based on?**

**Response:** Thank you for the opportunity to clarify. The 20% represents the percentage of the 46 participants who successfully presented to the CDO for program enrollment but were ultimately not enrolled into any scheme due to systems barriers, including that the CDO officer was too busy to attend to them, or that there was no available group for them to join. We have clarified these numbers in the text on page 13, edited figure 3, and edited the text included on lines 340-345 to read as below:

*“Even after successfully presenting to a development office for program enrollment, our survey results revealed that 20% of eligible participants were ultimately not enrolled into any program due to systems barriers, including strict limitations to enrollment based on the terms and structure of government-supported programs (e.g., there was no available group to join, or that the officer at the CDO was too busy to enroll them into a program).”*

9. **Lines 336 - 339: Appears to be new data that should be in the results section.**

**Response:** Thank you for this comment. These results were earlier reported in the results section on page 13, in the survey results section. However, we agree that they can be better summarized in the discussion section and have edited the text on lines 351-356 to read as follows:

***“****Other discrepancies noted in the survey, including mismatches between self-reported program enrollment versus actual enrollment based on logbook data, may reflect a breakdown in communication and lack of understanding among community members of the process of receiving social protection benefits.”*

10. **Discussion: The authors state their data did not provide reasons for the mismatch in supply and demand observed in address to social protection programs for those who were referred. Was any attempt made by investigators beyond their data collection to find out why a majority of patients referred for social program linkage were not enrolled or to provide a reason to those who were surveyed and reported not knowing why they did not get enrolled?**

**Response:** Thank you for this comment – yes, we conducted in-depth interviews with health center staff, community development officers, participants in the study, and with government stakeholders in an attempt to identify reasons for the drop-off between eligibility and enrollment. We are currently analyzing these data and plan to report the qualitative data in a follow-up manuscript, as it was beyond the scope of this current manuscript to include these results.

11. **References:**
    a. Line 448: Please provide a date when the online resource was accessed.

**Response:** Thank you, we have updated the citation to include the date of access.

b. Line 504: Please provide a date when the online resource was accessed.

**Response:** Thank you, we have updated the citation (now included as a reference in S1 File) to include the date of access.

**Reviewer #2**

1. **The authors addressed a very important problem that patients with tuberculosis and their families face related not only with the disease but related with social aspects including economic situation. There are some recommendations to improve this work and to make it suitable to be published.**

**Response:** Thank you for this positive comment. We have addressed some of the recommendations provided in the below responses and edited manuscript attached.

2. **In general although english is good, there are some typos and grammatical issues, that can be reviewed to improve the quality of the text.**

**Response:** We appreciate the opportunity to improve the manuscript grammatically and to fix any typos. We have reviewed the manuscript text in full and have fixed some tense errors, as well as some spacing and grammatical errors from the prior version. We have also edited several sentences throughout in response to reviewer comments and believe the newer version reflects results more clearly.

3. **In methods, it is not clear how was estimated the sample size for the survey, or what was the power of this sample.**

**Response:** Thank you for this comment. There were no formal calculations completed for the sample size for the survey included in this pilot study, as the main purpose was to collect supplementary data that could help explain some of the pilot results we observed. In lieu of formal sample size calculations, we worked with our local study team to understand what would be feasible to complete in the short 7 month pilot study period, and determined that approximately six participants per site per month (24 per month total) could be reasonably completed and would result in approximately 160 total surveys. We purposively selected study participants who agreed to participate in surveys based on gender and TB status. Ultimately, we were able to successfully contact 97 participants who ultimately completed the survey and provided us with sufficient supplementary data to help explain some of the pilot results.

4. **There are some analysis that could be proposed at least bivariate analysis even multivariable analysis, to better understand the relationship between some characteristics of the participants and actual access to social programs.**

**Response:** Thank you for suggesting these additional analyses. A comprehensive assessment of characteristics of individuals enrolled in our pilot linkage program was limited by the available data from clinical registers. Despite this, we did conduct some exploratory bivariate analyses of characteristics of participants in our survey (which is limited in sample size compared to overall enrollment) assessing differences across sex, age, multidimensional poverty, household size, bank account status, and employment status. We found that among survey participants, only age was associated with enrollment into a social protection program. Because this analysis is based only on survey participants, we did not include in the Results section as its generalizability to the full study population may be limited. We have added some text in the discussion section to reflect this limitation.

***“****In exploratory analysis of only those individuals who participated in our survey, we found that only age was associated with enrollment into a program, however as that analysis was of a small number of participants the results may not be generalizable.”*

5. **The authors describe a survey with three questions to identify who is eligible to receive support for social programs, however it is not clear how they choose these questions, or if this questionnaire is validated.**

**Response:** Thank you for the opportunity to clarify. This was not a validated questionnaire; rather, these three questions were used as a tool to conduct initial screening of patients undergoing TB diagnostic evaluation and were based on criteria established by the Government of Uganda when screening individuals seeking enrollment into government-supported social protection programs. First, community members are only eligible to receive benefits from CDOs that serve their subcounty of residence, therefore each client was screened to ensure he/she lived in a subcounty served by the CDOs included in the study. Second, government workers who are on payroll do not qualify for government-supported social protection program benefits, as they already receive permanent and pensionable employment. Third, those already benefiting from a government-supported SP program are ineligible for enrolling in additional programs. These criteria applied to all programs included in the evaluation and were simple for community health workers to administer quickly among many patients passing through the health center. We edited the text included in the manuscript on page 6 to indicate:

***“****After submission of their sputum sample, health center staff asked adults three questions to screen them for program eligibility; these questions corresponded to enrollment criteria established by the seven major government-supported social protection programs included in our pilot program. This included verification that the individual: 1) lived in a participating subcounty served by one of the included CDOs; 2) was not a salaried government worker; and 3) was not already receiving benefits from a government-supported social protection program.”*

6. **In results section, the tables 1 and 2 can be easier to understand if row titles do not repeat information that is included in first column, like n(%). Also, it would be desirable to include every category of the variables, because this allows to evidence that there was no missing data. Also for ordinal qualitative variables, it is recommended to present their categories in the corresponding order.**

**Response:** Thank you for these suggestions, which we have incorporated in edits to both tales 1 and 2 on pages 11, 12 and 13.

7. **Since a small number of programs were identified, they can be listed within the text and then refer to the Table for more information.**

**Response: Thank you, we have included the names of the programs on page 10 of the updated manuscript. The text now reads:**

***“****Briefly, these programs included: Youth Livelihood Program, Uganda Women Entrepreneurship Program, Emyooga, Parish Development Model, Social Assistance Grant for Empowerment, People with Disabilities, and Operation Wealth Creation. A more detailed description of the target population and intended function and structure of benefits and geographic coverage are summarized in* ***S1 Table****.”*

8. **It would be useful to have a cascade graphic to show how many patients were screened at the beginning and how many got the support.**

**Response:** Please see improved figure 1 which is included with then resubmission, which was edited in response to comment 10 in order to improve the quality and clarity of the figure.

9. **The table S2 has very important information that is recommended to include in results section and not like a supplementary table.**

**Response:** Thank you, we appreciate the reviewer’s encouragement regarding publication of this table which was uploaded as a supplementary file S2. Our rationale for providing this as a supplementary file was to present some of the important heterogeneity that we observed across the sites that were included in the study but were limited in that it was beyond the scope of this small pilot to determine the reasons for why these differences were present. In order to not distract from the main findings of the pilot, we decided to instead present these results in a supplementary file. As such, we would like for this table to remain in the supplement.

10. **Image quality of figure 1 could be improved to better show the results.**

**Response:** Thank you, we have uploaded a revised version of Figure 1 with the review response.

11. **In discussion section there are some data presented as result of the present study but those data where not described in the results section. Also it is recommended do not reference tables in this section.**

**Response**: Thank you for these suggestions, which we have accounted for in the edits to the discussion section in also our responses to reviewer #1. Please

12. **All references should be reviewed to fill the format requirements. Also within the text to unify the format of reference numbers with a space before the parentheses.**

**Response:** Thank you for the opportunity to review the reference formatting. We have reviewed all information referenced and have edited some of the references to be in compliance with the reporting style indicated in the PLOS Global Public Health Submission Guidelines. We have also revised the manuscript to include a space before the reference included in parentheses throughout.
