# Supplementary material for: Feasibility of a social protection linkage program for individuals at-risk for tuberculosis in Uganda
Source: PLOS Glob Public Health. 2023 Dec 8;3(12):e0002122. doi: 10.1371/journal.pgph.0002122 (PMC10707648; doi:10.1371/journal.pgph.0002122)
Supplement: Attachment — Submitted filename: PLOS GH Response to Reviewers_Oct23 pbs.docx [file pgph.0002122.s006.docx]

| UNIVERSITY OF CALIFORNIA, SAN FRANCISCO  berkeley • DAVIS • IRVINE • LOS ANGELES • RIVERSIDE • SAN DIEGO • SAn francisco |  | SANTA BARBARA • SANTA CRUZ |
| --- | --- | --- |

Priya Shete, M.D., M.P.H	Division of Pulmonary and Critical Care Medicine

Associate Professor of Medicine & Epidemiology	San Francisco General Hospital, Room 5K1

Co-Director, UCSF Implementation Science Training Program	1001 Potrero Avenue

Director, Collaborative on Pandemic Recovery and Readiness Research	San Francisco, CA 94110

Clinical Core Director, UCSF Center for Tuberculosis	Phone: 628-206-4694 | Fax: 628-206-8367

Email: [priya.shete@ucsf.edu](mailto:priya.shete@ucsf.edu)

October 16, 2023

Dear Dr. Gao and Editorial Colleagues,

Thank you very much for the opportunity to revise and resubmit our manuscript entitled “Feasibility of a social protection linkage program for individuals at-risk for tuberculosis in Uganda” (PGPH-D-23-01088) for consideration in PLOS Global Public Health. We are grateful for the additional reviews received and present below our responses and a summary of changes in response to Reviewer feedback.

We look forward to your response.

Sincerely,

Priya B. Shete, MD, MPH

**Review Comments to the Author**

**Reviewer #1**

1. **Thank you for addressing almost all of the comments raised on the first draft, however there are a few corrections that should be addressed:**

**Response:** Thank you for the opportunity to address your additional recommendations. Responses to these recommendations are below and the edited manuscript is attached.

2. **Line 239-240: The word "ineligible" is missing in this sentence.**

**Response:** Thank you for pointing out this error; we have added in “ineligible” to the sentence so now it reads correctly.

3. **Line 239-243: Suggest presenting proportions with numerator and denominator e.g. [134/855 (16%)] instead of n=134. This is recommended throughout where proportions are presented in text.**

**Response:** Thank you for this suggestion which we have implemented in the edited manuscript; in combination with the edit suggested above, the sentence now reads as follows:

*“Reasons participants were found to be ineligible included those living in a subcounty outside of the jurisdiction of the CDO (n=134/855; 16% of all screened), those not meeting age requirements (n=38/855; 4% of all screened), those who were government workers on payroll (n=14/855; 2% of all screened), and those already enrolled in social protection schemes (n=14/855; 2% of all screened).”*

4. **Table 1: Suggest labelling category as "Micro-bacteriologically confirmed TB" instead of "Diagnosed with..."**

**Response:** Thank you, we have edited the label in the table as suggested.

5. **Table 1: Suggest removing Treated category and presenting Treatment outcome only as: Not treated, Completed, Cured, Transferred out, Lost to follow up, Deceased. Please also include a description of the difference between completed and cured?**

**Response:** Thank you for this helpful suggestion to improve the Table results. We have done as suggested and removed the “Treated” rows, and added a row below the “Treatment outcome” category” to indicate the number of participants “Not treated”, and corrected the percentages provided following the number for each row. We also have added a footnote to Table 1 to explain the different categories for “Treatment outcome” and a reference for where these definitions were derived.

6. **Line 319: Suggest rephrasing to .."less than 20% of the 655 who were eligible..." and remove (n=655).**

**Response:** We agree with this edit to make this sentence read more clearly; in response to this comment and also a comment from reviewer number 2, we have edited the sentence to instead now read as follows:

***“****Among the total eligible persons linked to CDOs, less than 20% of those who were eligible to receive social protection benefits were enrolled into a program.”*

7. **Line 378-380: Suggest remove or rephrase this statement so that it doesn't sound like new information being presented in the discussion. Perhaps consider rephrasing as a recommendation for future research for the authors?**

**Response:** Thank you for this comment; we added in this additional sentence in response to a comment provided by Reviewer #2 in the last review: *“There are some analysis that could be proposed at least bivariate analysis even multivariable analysis, to better understand the relationship between some characteristics of the participants and actual access to social programs.”*

However, we agree with the suggestion to edit the presentation of these results so that new data are not being presented in the discussion. Thus, we have added in the “Survey results” section from lines 291-294 the following sentence:

*“In an exploratory analysis of only those individuals who participated in our survey, we found that only age was associated with enrollment into a program; on average, those enrolled were younger compared to those who were not (28.9 years versus 40.0 years, respectively).”*

Additionally, we edited the sentence in the discussion to now read:

*“Our exploratory analysis demonstrated that those enrolled were younger than those that were not; however as that analysis was of a small number of participants the results may not be generalizable.”*

**Reviewer #2**

1. **Thank you for your responses, most of the comments were addressed. However, there are still some aspects that could improve the work.**

**Response:** Thank you for the additional comments to improve the manuscript; we have provided responses to the comments provided below and attached to this response a revised manuscript that incorporates suggested edits.

2. **Regarding statistical analysis, it is not necessary to generalize results, but the relationship between characteristics can help, for example, to understand the relationship between sex and the result of a specific sample.**

**Response:** We agree that often bivariate or multivariate analyses can be helpful in interpreting results. We conducted bivariate analyses in our exploratory survey and reported the results in lines 307-310: *“In an exploratory analysis of only those individuals who participated in our survey, we found that only age was associated with enrollment into a program; on average, those enrolled were younger compared to those who were not (28.9 years versus 40.0 years, respectively).”*

3. **Furthermore, as the authors mentioned that this is a study to evaluate the results of implementation and enrollment, it is suggested to define the outcome as feasibility, which is a composite outcome, with indicators as described in line 198.**

**Response:** We agree with the reviewer’s suggestion and have included an additional sentence from 204-206 that reads: *“Taken together, these implementation outcomes were used to interpret the overall feasibility of the linkage program. In addition, we assessed technical feasibility of HC screening for program eligibility by comparing those who were found eligible at the HC to those found eligible at the CDO.”*

4. **Tables were improved, it is suggested in tables 1 and 2, for the last column title only to include the most frequent unit (example: n(%)) used in the table, and for those variables with a different unit of measurement to indicate it in the row next to the variable name.**

**Response:** Thank you for this suggestion to further improve the tables included in the manuscript; we have edited tables 1 and 2 as suggested.

5. **It is not clear how acceptability was assessed, which is mentioned several times.**

**Response:** Thank you for the opportunity to clarify. We reported on the acceptability of the linkage program in the results (lines 250-252) and discussion (lines 321-322), but did not include a clear definition in the “Outcomes” section. This has been amended in the revised manuscript from lines 208-209 and now reads:

***“****The proportion of those who were eligible for a program, interested in being referred and ultimately accepted a referral to a CDO was used to assess linkage program acceptability.”*

6. **It is not recommended to refer to tables from results in the discussion.**

**Response:** Thank you. We have edited some of the discussion section to remove specific numbers already reported in prior sections of the manuscript, in particular from lines 327-332.

7. **Although the text has been improved, there are still some spaces missing before reference parenthesis.**

**Response:** We have reviewed the manuscript in its entirety and have made the corrections to the missing spaces; we thank the reviewer for this attention to detail.

8. **Abstract results can be revised to make them clearer, for example how many participants were interviewed.**

**Response:** Thank you, we agree with the suggestion to make the abstract results clearer. Specifically, we have edited the results section from lines 42-49 to read as follows:

***“****In surveys conducted among 97 participants, 46 of the 60 (77%) people who reported that they sought enrollment at the CDO were not enrolled into a social protection program. Reasons provided for non-enrollment among these 46 participants were either unknown (n=25, 54%) or due to operational challenges at the CDO including a lack of human resources or available groups to join in the social protection program (n=20, 43%). 61 survey participants (63%) indicated that they would not have sought social protection enrollment without the referral program.”*
